# Generative Artificial Intelligence–Driven Clinical Case Simulation in Temporomandibular Disorder Education: ChatGPT Versus Real Patients

**DOI:** 10.1002/jdd.70104

**Published:** 2025-11-12

**Authors:** Paula Rodrigues‐Pereira, Maria Amália Pereira Dias‐Calças, Alex Moreira Mélo, Melissa de Oliveira Melchior, Lucas Gaspar Ribeiro, Antônio Pazin‐Filho, Jardel Francisco Mazzi‐Chaves, Laís Valencise Magri

**Affiliations:** ^1^ Departamento de Odontologia Restauradora Faculdade de Odontologia de Ribeirão Preto Universidade de São Paulo (FORP/USP) Ribeirão Preto São Paulo Brazil; ^2^ Departamento de Clínica Médica Faculdade de Medicina de Ribeirão Preto Universidade de São Paulo (FMRP/USP) Ribeirão Preto São Paulo Brazil

**Keywords:** artificial intelligence, ChatGPT‐3.5, clinical simulation, dental education, orofacial pain, temporomandibular disorders

## Abstract

**Background:**

Temporomandibular disorders (TMDs) and orofacial pain (OFP) demand advanced diagnostic and clinical reasoning skills in dental education. Traditional simulations with real patients face limitations in availability and standardization. Generative artificial intelligence (GAI), such as ChatGPT‐3.5, has emerged as a potential tool for clinical training.

**Methods:**

This blinded, cross‐sectional, crossover study involved 30 undergraduate dental students, each completing two simulated cases: one with ChatGPT‐3.5 and one with standardized real patients. Cases were developed and validated by TMD/OFP specialists via the Delphi method and incorporated into the AI through structured prompts. Quantitative parameters (such as responses, word count, follow‐up questions, reformulations, and diagnostic accuracy) and qualitative aspects (such as empathy, clarity, and relevant information) were analyzed.

**Results:**

GAI simulations provided higher information density (231 vs.167 relevant units; *p *< 0.001) and clearer reasoning flow. Students interacting with real patients asked more follow‐up questions (*p *= 0.004) and required more reformulations (*p* = 0.011), indicating more adaptive communication. Diagnostic accuracy did not differ significantly (*p *> 0.05). Relevant information correlated positively with diagnostic accuracy (*r* = 0.484; *p* = 0.007), whereas total word count correlated negatively (*r* = −0.386; *p* = 0.035).

**Conclusions:**

ChatGPT‐3.5 matched real patient simulations in diagnostic reasoning for TMD/OFP. Combining GAI's scalability and standardization with real patient variability may optimize clinical competency training.

## 1 Introduction

Temporomandibular disorders (TMDs) are currently understood as a set of signs and symptoms that characterize a musculoskeletal pain syndrome associated with multisystemic alterations, as well as behavioral, emotional, and social interaction changes, recognized as manifestations of central nervous system dysregulation [1, 2, 3]. Major predictors for the development of TMD include the presence of comorbidities, non‐painful orofacial symptoms (e.g., self‐reported parafunctions), high frequency of somatic symptoms, poor sleep quality, and genetic and epigenetic factors. The most common signs and symptoms are orofacial pain (OFP), joint sounds, and alterations in mandibular mobility [2, 4].

In undergraduate and postgraduate education, the teaching of TMD and OFP aims to provide students with the ability to perform a thorough anamnesis and physical examination, which includes palpation of the craniofacial muscles, evaluation of mandibular opening, and detection of joint sounds, ultimately reaching a diagnosis based on current classification criteria, such as the Diagnostic Criteria for Temporomandibular Disorders or the International Classification of Orofacial Pain [5, 6, 7]. It is important to note that the complexity of educational content differs between undergraduate and postgraduate levels. At the undergraduate level, teaching TMD is considered essential and aligns with the new standards of the Commission on Dental Accreditation (CODA), which mandate the inclusion of core competencies in OFP within the dental curriculum. In postgraduate education, however, the depth and emphasis of TMD and OFP training vary according to each specialty discipline, ranging from prosthodontics to dedicated OFP programs. In addition to diagnostic training, students across both levels are also instructed to develop skills and competencies related to history‐taking and clinical reasoning.

The use of generative artificial intelligence (GAI), such as ChatGPT, in healthcare education represents an innovative and promising approach. In addition to ChatGPT, other conversational agents have been explored in medical and dental education, including IBM Watson Assistant, used for interactive clinical decision support, and Ada Health, a symptom‐assessment chatbot designed to simulate patient triage. These examples illustrate the broader landscape of conversational AI tools with potential applications in health professions education. Recent studies demonstrate the potential of this technology to enable realistic and interactive simulations that complement traditional clinical learning. The integration of AI into educational environments offers opportunities for the development of practical skills and improved clinical decision‐making [8, 9, 10]. Moreover, interacting with AI‐based chatbots can enhance students’ ability to handle complex and varied situations, including the dentistry field [11, 12]. Therefore, incorporating GAI into clinical simulations is expected to provide healthcare students with practical experience and evidence‐based decision‐making skills, better preparing them for clinical practice.

This new generation of AI can produce complex outputs, providing natural language feedback, generating reports and summaries by utilizing its database during simulated patient interviews [13, 14]. As a result, the potential use of ChatGPT‐3.5 in patient triage and clinical assessment has been the subject of several studies [8, 9, 10, 11, 12, 13, 14, 15, 16]. The literature on artificial intelligence in dentistry highlights both the opportunities and challenges associated with its implementation. AI has been applied in multiple facets of dentistry, ranging from imaging diagnostics to patient management and clinical decision‐making. However, these advancements come with significant challenges, such as the need for high‐quality datasets to train AI algorithms, ethical concerns related to patient privacy, and issues regarding legal responsibility [17, 18]. A conceptual framework for clinical reasoning in dentistry emphasizes its fundamental importance to dental practice and education [19, 20]. Based on these considerations, the present study seeks to critically explore how AI can be effectively leveraged in dentistry while responsibly addressing the challenges associated with its implementation, aiming to enhance professionals’ clinical reasoning and the quality of dental care delivered to patients.

In light of these considerations, the present study hypothesizes that, among undergraduate dental students, (1) simulations conducted with GAI will yield results comparable to those conducted with real patients in developing clinical competencies related to TMD/OFP; (2) the use of GAI will present advantages, such as greater accessibility and flexibility for educational simulations, but also limitations regarding emotional expressiveness and clinical variability compared to real patients; and (3) the responses generated by GAI will be equivalent to those of real patients in terms of quality, consistency, and clinical appropriateness for teaching TMD/OFP, enabling students to develop competencies similarly. This study seeks to investigate the applicability of GAI, specifically ChatGPT‐3.5, as a clinical simulation tool in TMD/OFP education. Its aim is to compare simulations using GAI with those involving real patients through quantitative and qualitative parameters and to identify potential limitations and advantages of using GAI for educational purposes. This investigation addresses a critical gap in the literature and has the potential to inform future strategies for integrating GAI into health professional education.

## 2 Materials and Methods

### 2.1 Ethical Considerations

The Research Ethics Committee of the School of Dentistry of Ribeirao Preto (FORP/USP) approved this study (CAAE: 80542424.2.0000.5419). All participants received detailed information about the study procedures and voluntarily decided whether to participate. Students were explicitly informed that their participation would not be associated with grades in the TMD/OP course. Those who agreed to participate signed a written informed consent form before inclusion in the study.

### 2.2 Sample Composition

This was an exploratory study based on a convenience sample of 30 participants. The sample size was defined pragmatically, considering logistical feasibility and the availability of eligible students who had previously completed the TMD/OP course. No formal sample size calculation was performed, as the study was not designed to test a specific hypothesis with predetermined power, but rather to generate preliminary evidence regarding the applicability of GAI in TMD/OFP simulation‐based education. The crossover design allowed each participant to experience both simulation models, increasing the internal validity and the efficiency of comparative analyses. The sample of undergraduate dental students was recruited from the School of Dentistry of Ribeirao Preto (FORP/USP. Eligibility criteria included prior completion of the TMD/OP course, which is offered during the seventh semester of the program, and demonstration of sufficient computational, visual, and cognitive skills to interact effectively via typed text. Exclusion criteria encompassed any history of mental or emotional health conditions that could interfere with interaction with ChatGPT or the ability to cope with simulated stressful situations, as well as any other medical condition or circumstance that might impair effective participation in the simulations. Students with limited proficiency in Portuguese were also excluded.

### 2.3 Study Design

This study adopted a blinded, cross‐sectional, simulation‐based experimental design with a crossover model involving both GAI and standardized real patients. Two distinct clinical cases were utilized: one involving TMD of the myofascial pain with referral type, and the other involving odontogenic pain of the irreversible pulpitis type, as defined by the International Classification of Orofacial Pain [7, 21]. Each case was simulated using both real patients and the ChatGPT‐3.5 platform.

**FIGURE 1:**
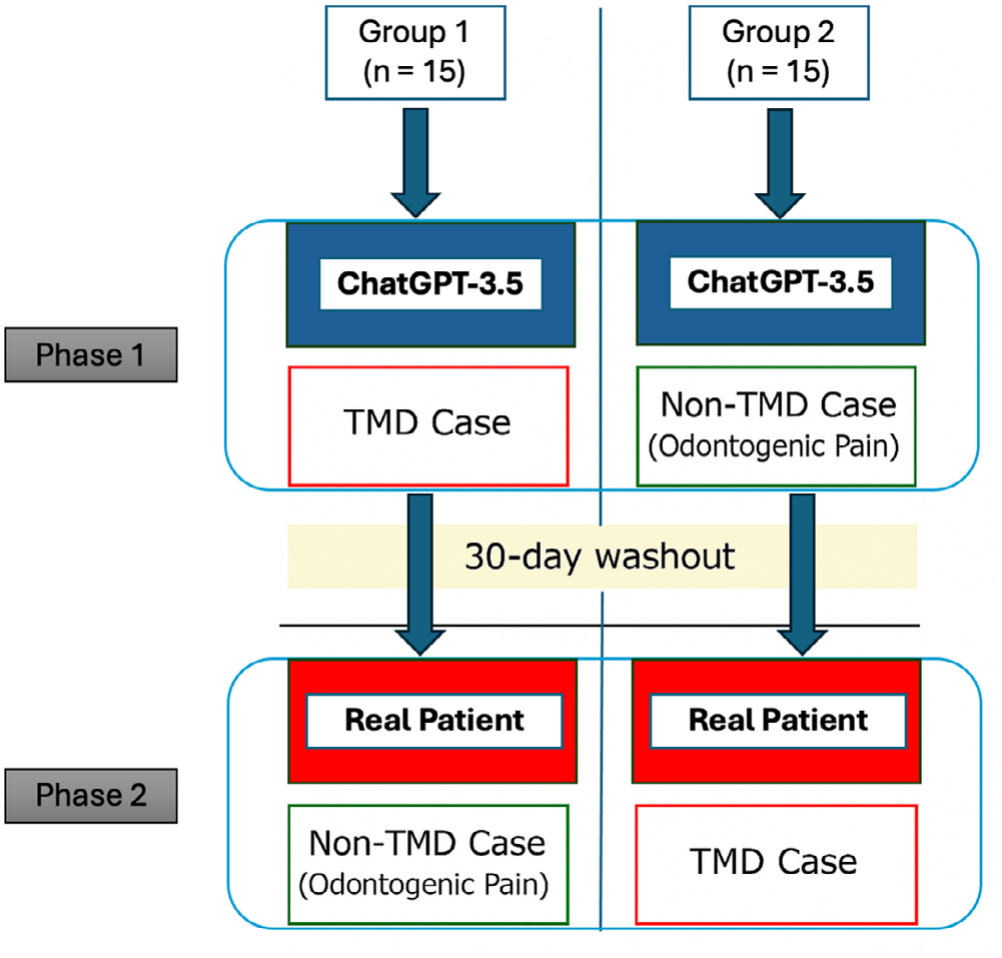
Crossover design of the study. In Phase 1, Group 1 (*n *= 15) interacted with ChatGPT‐3.5 and Group 2 (*n *= 15) with a real patient. Each group managed both a TMD case and a non‐TMD case (odontogenic pain). After a 30‐day washout, the simulation models were crossed over between groups so that all participants experienced both interaction modalities and both clinical scenarios.

Students participated in the computer‐based simulations under blinded conditions, meaning they were not informed whether they were interacting with a real patient or ChatGPT‐3.5. To ensure that all students were exposed to both interaction models, a crossover design was implemented: students who initially participated in the GAI simulation subsequently engaged with the real patient simulation, and vice versa. Participants were randomly allocated into two groups (Group 1 and Group 2, *n* = 15 each) through simple randomization (lottery method). In Phase 1, Group 1 interacted with ChatGPT‐3.5, while Group 2 interacted with a real patient. Both groups were exposed to two clinical scenarios — a TMD case and a non‐TMD case (odontogenic pain). After a 30‐day washout period, the groups crossed over so that each participant experienced the opposite simulation model in Phase 2 (Figure 1). This design ensured that all participants completed both simulation modalities (AI‐based and real patient) and both case types, while maintaining blinding regarding the simulation model being used. Blinding was deliberately incorporated to ensure that students approached both simulations with equivalent levels of attention and seriousness, thereby minimizing potential biases that could compromise the authenticity of the interactions and the internal validity of the findings.

### 2.4 Briefing, Simulation, and Debriefing Procedures

After students agreed to participate in the study, a simulation date was scheduled according to their availability and that of the research team. Each student participated in the simulation individually, with a total duration of approximately 1 h. Simulations were conducted in a closed room equipped with a computer and a timer to monitor the duration of each session. Students were instructed not to use cell phones and to remain focused throughout the activity. A member of the research team remained in the room during the entire simulation to control the timing and manage any unforeseen events. Before beginning the simulation, the informed consent form was read aloud, signed by the student upon agreement, and a copy was provided. Standardized briefing instructions were then delivered, ensuring consistency for all participants.

### 2.5 Briefing

The briefing phase represented the initial stage of the simulation, during which students were clearly informed about the objectives of the task and the activities they would be expected to perform, corresponding to the clinical scenario. All instructions were delivered in a detailed and standardized manner to ensure consistency across participants.

- Each participant underwent two simulation sessions, each with a maximum duration of 20 min. If the simulation was completed before the allotted time, students were instructed to inform the researcher present in the room.
- The simulations were conducted using a shared Google Docs document, which was already open on the computer available in the simulation room.
- During the simulations, students were required to assume the role of a dentist interacting with a patient. Each time a question was typed into the Google Docs document, a corresponding response from the “patient” appeared below.
- This question‐and‐answer exchange continued sequentially, replicating the flow of a natural patient–clinician interaction.
- In the final 5 min of each simulation, students were required to formulate a diagnostic hypothesis and propose one or more appropriate therapeutic management strategies. The researcher in the room notified the student when only 5 min remained.
- Upon completion of each simulation, participants were required to complete a Google Forms questionnaire to report their experience. This form included a question asking whether, in the participant's opinion, the interaction had been conducted with ChatGPT‐3.5 or with a real patient. This information was used solely for complementary purposes and was not included in the study's primary analyses, as it was unrelated to the research objectives.
- The second simulation session was conducted following the same standardized protocol as the first.
- In the event of any questions or technical issues, the researcher present paused the timer, resolved the issue, and resumed the simulation once it was addressed.
- At the end of both simulations, the researcher disclosed which interaction had involved the real patient and which had involved ChatGPT‐3.5.

### 2.6 Simulations

The simulation was conducted using a computer with access to a shared Google Docs file. Each student interacted exclusively through written text within this document, which was shared at the start of the simulation. The document was simultaneously accessible to the participant, the standardized real patient, or the researcher replicating ChatGPT‐3.5 responses (located in a separate room), and a researcher who monitored the interaction. The interaction workflow varied according to the assigned simulation group:

- **ChatGPT‐3.5 interaction**: A member of the research team, with access to the shared document, copied each question submitted by the student in real time and pasted it into ChatGPT‐3.5 after entering the simulation prompt (described in detail below). The AI‐generated response was then copied and pasted into the shared document for the student to view.
- **Real patient interaction**: A standardized real patient, also with access to the shared document, responded directly and in real time to the questions submitted by the student.

Each student participated in two simulations, one with ChatGPT‐3.5 and one with a real patient, in sequential order and under blinded conditions. The student remained in a separate room from the individual (researcher or real patient) providing the responses, thereby preventing identification of the simulation model.

A time limit was imposed to replicate realistic clinical practice, where clinicians often need to make decisions under time constraints. Time management is an essential competency to be developed during dental education, and a 20‐min limit per simulation was established to balance the realism of the scenario with pedagogical effectiveness. Additionally, this time limit ensured the feasibility of the study, allowing all participants to complete both simulations within a controlled period, thereby facilitating data collection and standardizing evaluation conditions. This approach was particularly important for ensuring comparability of results and minimizing variables that could influence student performance.

In the final 5 min of each simulation, students were required to assign a dichotomous diagnostic hypothesis, choosing between “TMD case” and “non‐TMD case,” and to propose one or more therapeutic management strategies appropriate to the case. The researcher presented in the room and notified the participant when 5 min remained.

An additional methodological strategy was implemented to reduce potential bias: responses from both ChatGPT‐3.5 and the real patient were not typed directly into the shared document by the responder. This avoided differences in typing speed and the appearance of text on the screen, which could have led students to perceive differences in interaction style and infer which simulation model they were experiencing. This design ensured that the student's experience with both simulation models remained as uniform as possible.

### 2.7 Clinical Cases

#### 2.7.1 Real Patients

A real patient with preserved communication skills (verbal and cognitive abilities) was previously selected based on an initial anamnesis interview. This patient presented with TMD of the myofascial pain with referral subtype. Subsequently, another patient with odontogenic OFP diagnosed as irreversible pulpitis, according to the International Classification of Orofacial Pain, was selected from the emergency service at FORP/USP after initial care [7, 21].

These patients were thoroughly briefed about the simulation procedures and instructed to respond clearly, sincerely, and objectively to all questions posed by the students. If faced with a question they were unable to answer, they were advised to state simply that they did not know or did not have the necessary information. Under no circumstances were students provided with any identifying information about the patients.

The researcher responsible for replicating the responses from either the real patients or the GAI (ChatGPT‐3.5) was calibrated with respect to response timing to avoid revealing the source of information. When the responses were generated by ChatGPT‐3.5, the researcher copied the AI's answer and pasted it into the shared interaction document. When the responses were provided by the real patients, the researcher transcribed the exact patient statement into a separate document and subsequently copied and pasted it into the shared document, following the same procedure used for GAI responses. This approach was chosen to eliminate differences in typing speed and the appearance of text on the screen, which could otherwise have led students to identify which simulation model they were interacting with.

### 2.8 Simulated Cases (Interaction via ChatGPT‐3.5)

#### 2.8.1 Development of the Simulated Cases

Two simulated cases were developed: one representing a painful TMD (myofascial pain with referral subtype) and the other representing odontogenic OFP (differential diagnosis of TMD). The development process followed the Delphi method [22], prior to the student simulations, and included the following steps:

- Selection of a panel of experts (three specialists in TMD and OFP with more than 5 years of clinical experience).
- Initial contact with the experts, invitation to participate in the study, and collection of signed informed consent forms.
- Joint construction of the two clinical cases: the TMD case was developed according to the Diagnostic Criteria of the DC/TMD, and the odontogenic OFP case according to the criteria of the International Classification of Orofacial Pain [7, 21]. The simulation prompt to be used with ChatGPT‐3.5 was also constructed in this stage.
- Simulation Round 1: experts piloted the clinical cases.
- Collection and analysis (qualitative and quantitative) of all questions and responses from Simulation Round 1.
- Provision of structured feedback to the expert panel based on the analyses.
- Simulation Round 2: experts re‐evaluated the refined cases.
- Collection and analysis (qualitative and quantitative) of the data from Simulation Round 2.
- Finalization of the process and drafting of the final report, which included the validated clinical cases and insertion prompts, as well as the gold‐standard sequence of expected questions and responses. This reference standard was subsequently used for comparative analysis with student performance.

## 3 Clinical Case Descriptions

*TMD Case—Myofascial Pain with Referral*: A 45‐year‐old female teacher presented with severe cheek pain when chewing, both intra‐ and extra‐oral, localized in the region of tooth #16, with a 2‐month history. She reported no improvement with analgesics and worsening of pain during stressful situations. The pain had intensified in the past week following a conflict with a student. Clinical and imaging examinations revealed that tooth #16 had previously undergone endodontic treatment and presented no carious, periodontal, or periapical lesions. Palpation of the right masseter muscle elicited pain referred to the region of tooth #16. There was no limitation of mandibular opening or lateral excursions, and no joint sounds were detected in the TMJs. Abfraction lesions were observed on teeth #14–17, with associated cold sensitivity, except for tooth #16. The patient had a prior medical history of depression, migraine, and fibromyalgia, and was taking duloxetine 30 mg daily.
*Non‐TMD Case—Odontogenic OFP (Irreversible Pulpitis)*: A 45‐year‐old male truck driver presented with severe throbbing pain in the left zygomatic region radiating to the ear, with onset 2 days prior. The pain was non‐responsive to analgesics and worsened when lying down. Extraoral clinical examination failed to reproduce the pain on palpation. A joint click was noted in the left TMJ. Intraoral examination of Quadrant 2 revealed positive pulp vitality tests for all teeth, with pain exacerbated on tooth #27. This tooth also presented with an unsatisfactory restoration. A missing lateral incisor was noted. The patient had a prior medical history of chronic low back pain and sleep apnea.

## 4 Final Standardized Prompt

The final standardized prompt used with ChatGPT‐3.5 was refined and validated by the expert panel to ensure reproducibility and consistency across all simulations. The prompt instructed the AI to act strictly as the patient, providing information only when asked by the student, without offering diagnoses or treatments. The full prompt is presented below:

*Prompt*: “You are simulating a standardized patient in a clinical interview with a dental student. Your role is to faithfully reproduce the case description provided, which corresponds to a validated clinical scenario of OFP. Your task is to act only as the patient: answer the student's questions exactly as the patient would, based on the clinical history and details included in the case description. Do not add information beyond what is described in the case, do not invent new symptoms or personal data, and do not omit key findings provided. Do not provide explanations, diagnoses, or treatment suggestions under any circumstances. Respond in a natural and realistic way, as if you were a real patient. Keep your answers concise and coherent with the case, but you may occasionally include brief hesitations, emotional expressions, or everyday language to enhance realism (e.g., “I'm not sure,” “It bothers me when I chew,” “This pain makes me feel anxious”). Avoid technical or professional terminology. Always wait for the student to ask questions before providing information. Do not anticipate details unless specifically asked. Your answers should be consistent, reproducible, and limited strictly to the clinical scenario. If a student asks something not relevant to the case or not included in the description, answer realistically with a neutral response such as: “I don't know” or “I haven't noticed that.” Your goal is to allow the student to practice history‐taking, hypothesis generation, and clinical reasoning, in conditions comparable to an interaction with a real patient, while maintaining fidelity to the validated scenario.”

### 4.1 Debriefing

At the end of each simulation, students were required to complete a Google Forms questionnaire designed to capture their experience with the simulation. This analysis was exploratory and complementary in nature and was not directly related to the primary aim of the study. In this questionnaire, students were asked, based on their perception, whether they believed the interaction had been conducted with ChatGPT‐3.5 or with a real patient. The questionnaire included the following items:

- “Based on your experience in this simulation, what is your level of satisfaction on a scale from 0 to 10 (0 = not satisfied, 10 = extremely satisfied)?” (Closed‐ended question, response options from 0 to 10).
- “What were the strengths and the areas for improvement in this simulation experience?” (Open‐ended question requiring detailed responses).

The primary purpose of the questionnaire was to address the secondary aim of the study, which was to identify the potential advantages and limitations of using GAI in clinical case simulations in the field of TMD and OP, particularly with respect to student interaction with each simulation model. After completion of data collection, students were invited to a collective debriefing session. In this session, the main findings from the simulations were presented, along with the knowledge gaps identified in the establishment of dichotomous diagnostic hypotheses (TMD vs. non‐TMD) and therapeutic management strategies.

### 4.2 Statistical Analysis

The data analysis was conducted using an integrated approach, combining quantitative and qualitative procedures. Quantitative data were analyzed using JASP software (version 0.18.3, University of Amsterdam). Descriptive analyses were first performed to characterize continuous variables (mean, standard deviation, median, and interquartile ranges) and categorical variables (absolute and relative frequencies). Subsequently, the groups (GAI vs. real patients) were compared regarding the total number of responses, total number of words, frequency of additional interactions (follow‐up questions), and number of response reformulations using parametric or non‐parametric tests, depending on data distribution. To compare the percentages of correct and incorrect dichotomous diagnostic hypotheses (TMD case vs. non‐TMD case), Fisher's exact test was applied, with a significance level set at 5% (*p* < 0.05). In addition, point‐biserial correlations were performed between diagnostic accuracy and the quantitative and qualitative variables to assess the association between students’ performance and the evaluated parameters.

Qualitative analyses were conducted using Bardin's Content Analysis method with the support of NVivo software (version 14, QSR International) [23, 24]. The transcribed responses from the simulators (ChatGPT‐3.5 and real patients) underwent a three‐stage thematic categorical analysis: (1) pre‐analysis, with an initial overview of the material; (2) exploration of the material, with coding and categorization of keywords and expressions related to the predefined constructs (empathy, clarity in communication, and relevant information); and (3) treatment of the results, with qualitative inferences regarding the depth, frequency, and communicational adequacy of the responses. Additionally, the typology of students’ questions (open vs. closed) and the evolution of clinical reasoning were analyzed, allowing for a comparison of similarities and differences between simulations with GAI and real patients.

## 5 Results

Table 1 presents a comparative analysis of key quantitative parameters derived from the simulated clinical interactions using ChatGPT‐3.5 and real patients. The average number of responses per interaction was slightly higher in the real patient simulations (6.1 ± 1.5) compared to ChatGPT (5.3 ± 1.2), indicating a more dynamic dialogic exchange in human‐mediated encounters. Although the total word count per case was marginally higher in ChatGPT simulations (193 ± 28 vs. 179 ± 34), this may reflect the model's tendency to provide complete and structured responses without requiring additional prompts. Notably, students asked a greater number of follow‐up questions when interacting with real patients (6.7 ± 1.8) than with ChatGPT (4.6 ± 1.4), suggesting that real patient simulations elicited more exploratory and investigative communication. Similarly, the need for answer reformulations—indicative of misunderstandings or insufficient initial clarity—was nearly twice as high in interactions with real patients (1.5 ± 0.9) compared to ChatGPT (0.8 ± 0.7). These findings underscore distinct communicative dynamics between the two simulation modalities and suggest that real patient simulations may encourage more active clinical reasoning and iterative information gathering.

**TABLE 1:** Quantitative comparison between simulations with generative artificial intelligence (ChatGPT‐3.5) and real patients.

Evaluated criterion	ChatGPT (mean ± SD)	Real patient (mean ± SD)	*p *value
Number of responses per interaction	5.3 ± 1.2	6.1 ± 1.5	0.081
Total word count per clinical case	193 ± 28	179 ± 34	0.112
Additional questions asked by students	4.6 ± 1.4	6.7 ± 1.8	0.004*
Reformulations of answers by the simulator	0.8 ± 0.7	1.5 ± 0.9	0.011*

*Note*: Mean and standard deviation were calculated based on 15 simulated interactions per clinical case type. “Additional questions” refer to follow‐up inquiries made by students during the interaction. “Reformulations” indicate how often the simulator had to adjust or repeat an answer due to lack of immediate comprehension.

*p < 0.05.

**FIGURE 2:**
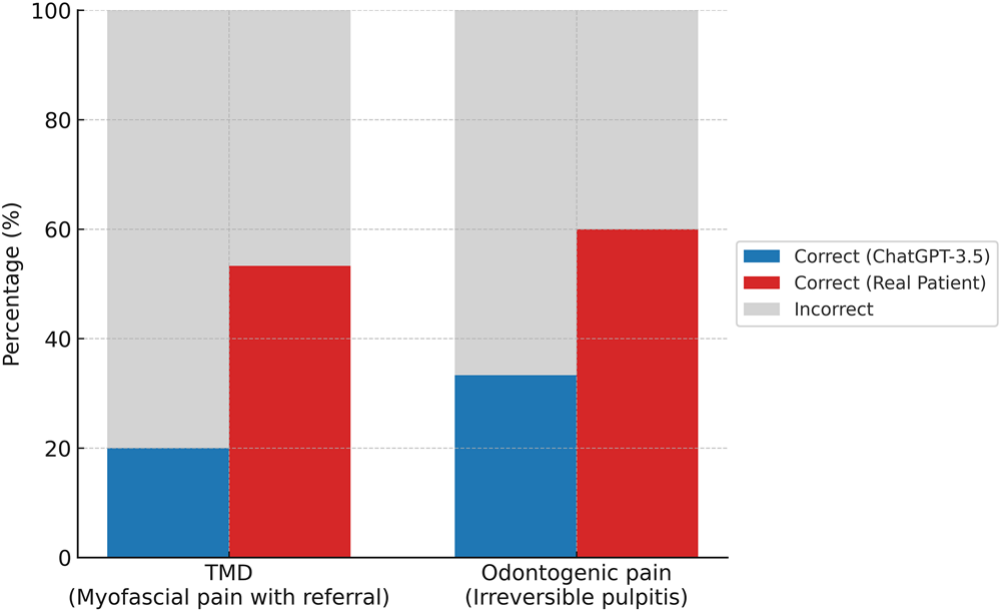
Percentage of correct and incorrect initial diagnostic hypotheses—myofascial pain with referral (TMD) and irreversible pulpitis (odontogenic pain)—comparing simulations performed with generative artificial intelligence (ChatGPT‐3.5) and with a real patient. Each bar represents the proportion of students who correctly (colored) or incorrectly (gray) identified the initial diagnosis in each clinical condition.

Figure 2 displays the diagnostic accuracy of students in formulating initial hypotheses for two simulated clinical cases: myofascial pain with referral (TMD) and irreversible pulpitis (odontogenic pain). In the TMD case, a higher percentage of correct responses was observed in the real patient simulation (53.3%) compared to ChatGPT‐3.5 (20%). Similarly, for the odontogenic pain case, students performed better with the real patient (60%) than with the AI‐based simulation (33.3%). These findings suggest that, while generative AI may serve as a useful tool for teaching clinical reasoning, its ability to convey relevant diagnostic cues remains limited when compared to real patient interactions.

The point‐biserial correlation analysis revealed statistically significant associations between diagnostic accuracy and specific variables assessed in the clinical simulations. The inclusion of relevant clinical information in responses showed the strongest positive correlation with diagnostic accuracy (*r* = 0.484; *p* = 0.007), indicating that clinically pertinent content plays a key role in supporting students’ diagnostic reasoning. Conversely, the total number of words was negatively correlated with accuracy (*r* = −0.386; *p* = 0.035), suggesting that longer responses do not necessarily improve diagnostic precision. Although variables such as empathy (*r* = 0.299; *p* = 0.108) and clarity in communication (*r* = −0.068; *p* = 0.720) did not reach statistical significance, the observed trends may warrant further investigation in future studies with greater statistical power.

The qualitative analysis, based on Bardin's Content Analysis, revealed relevant differences between simulations performed by ChatGPT‐3.5 and those with a real patient regarding the categories of empathy, clarity in communication, and relevant information (Table 3). ChatGPT demonstrated a higher frequency of expressions related to empathy (*n* = 67) compared with the real patient (*n* = 32), although these were more formal and standardized, whereas the real patient exhibited genuine empathy, tied to lived experiences and a greater emotional charge. Regarding clarity in communication, ChatGPT also showed more occurrences (*n* = 30 vs. *n* = 16), characterized by more structured and didactic responses, while the real patient, despite being spontaneous, often displayed hesitations and difficulty organizing speech. The category relevant information was the most prevalent in both groups, being substantially higher in ChatGPT (*n* = 231) than in the real patient (*n* = 167), with more complete, objective, and organized responses. On the other hand, the real patient's responses presented greater subjective contextualization, adding emotional nuances and situational details that can be valuable for clinical reasoning. These results suggest that, despite the higher informational density observed in ChatGPT‐3.5, interactions with real patients still provide greater authenticity and emotional complexity, which are essential elements for the development of students’ communication skills.

The analysis of questions formulated by students after the simulations (Table 4) revealed important differences between those exposed to ChatGPT‐3.5 and the real patient. Students who interacted with ChatGPT asked a higher proportion of open questions (58%) compared with those who interacted with the real patient (48%). Open questions allow for more detailed and narrative information, and in the ChatGPT group, they were more organized and structured. Closed questions were slightly more frequent in the real patient group (52% vs. 42%), generally used to confirm pre‐existing hypotheses. Regarding the evolution of clinical reasoning, students in the ChatGPT group demonstrated a clearer logical progression, beginning with general questions (onset, duration, pain characteristics) and advancing to more specific diagnostic hypotheses (TMD vs. odontogenic pain). In contrast, the group interacting with the real patient showed greater spontaneity but less organized alternation between open and closed questions, which may hinder diagnostic integration. These findings suggest that ChatGPT‐3.5, by providing information in a structured and consistent manner, favors the sequential development of students’ clinical reasoning.

Table 5 provides a detailed comparison between clinical simulations conducted with GAI (ChatGPT‐3.5) and real patients, highlighting the main advantages and limitations of each model. GAI‐based simulations proved to be superior in terms of information density, offering more complete and organized responses (231 vs. 167 relevant information units), as well as greater standardization and accessibility, allowing multiple simulations to be performed in any educational context. However, these simulations exhibited important limitations, such as reduced clinical variability, absence of non‐verbal cues, and empathy perceived as less genuine, with more linear and predictable responses. Conversely, real patient simulations were characterized by greater authenticity and emotional richness, natural clinical variability, and the presence of non‐verbal cues, which are essential for clinical practice. Nevertheless, they showed lower information density, higher unpredictability that may challenge less experienced students, and issues related to standardization and participant availability. These findings demonstrate that each simulation model has complementary strengths and limitations that can be strategically combined in teaching clinical competencies in TMD/OP.

## 6 Discussion

This study investigated the applicability of GAI, through ChatGPT‐3.5, as a simulation tool for teaching clinical competencies in TMD and OFP, comparing it with real patient simulations. The main findings revealed that GAI‐based simulations demonstrated greater information density, providing more relevant information units (231 vs. 167), as well as greater standardization, accessibility, and a clearer progression of clinical reasoning. Conversely, real patient simulations were characterized by greater authenticity and emotional richness, natural clinical variability, the presence of non‐verbal cues, and spontaneity in interactions, all of which are essential for clinical practice. Additionally, students’ diagnostic accuracy did not significantly differ between the two models, suggesting that GAI can offer a comparable experience to real patients in the development of diagnostic skills, albeit with distinct communicational characteristics. These results highlight the complementarity between the models and suggest that integrating both approaches may enhance learning in TMD/OP.

These results highlight the complementarity between the models and suggest that integrating both approaches may enhance learning in TMD/OP. It is also important to emphasize that this pedagogical approach is primarily suitable for knowledge‐based training and the assessment of clinical reasoning skills. The use of text‐based generative AI simulations is not intended to replace practical training in procedural or technical competencies, such as physical examination or clinical interventions. Therefore, its main contribution lies in supporting the development of diagnostic reasoning and communication strategies, while procedural skills must continue to be taught through hands‐on clinical practice.

Although the overall diagnostic success rate was significantly higher with real patients compared to AI, the average rates (53% for dental cases and 60% for TMD cases) were relatively low considering that the required task was only to distinguish between TMD and non‐TMD. Several factors may have contributed to this finding. First, the students may not have been optimally prepared for the exercise, as their didactic course might have been completed sometime before the simulation without a refresher session. Second, the text‐based nature of the simulation may have limited the richness of interaction, as non‐verbal cues, physical examination, and spontaneous conversational dynamics were absent. These factors may have influenced the accuracy of students’ diagnostic reasoning. This should be acknowledged as a limitation of the study, reinforcing the need for integrating simulation exercises with ongoing didactic reinforcement and for exploring multimodal AI interfaces in future studies.

Undergraduate education in TMD and OFP requires a multifaceted approach that goes beyond the mere transmission of academic content. In this context, it is essential to understand and articulate the concepts of content, skill, attitude, and competence, which are fundamental to students’ professional development. Content is central to the educational process, but its effective internalization requires meaningful interaction with the student's prior knowledge. In the context of TMD and OFP, this involves not only theoretical understanding of the conditions but also the ability to apply such knowledge in clinical practice, demonstrating clinical skills and technical dexterity [25]. Furthermore, positive attitudes toward patients and the pursuit of continuous improvement are essential to training ethical professionals committed to patient well‐being [26]. Competence, in turn, involves the harmonious integration of these elements, enabling graduates to face complex challenges with confidence and effectiveness [27].

Clinical case simulations, both real and simulated, have emerged as powerful tools in health professional education, allowing students to practice clinical skills and decision‐making in controlled and safe conditions [28]. In the context of TMD and OFP, simulation offers a platform for the practical application of theoretical knowledge, allowing students to develop competencies related to history‐taking and clinical reasoning. It is worth noting that the potential of GAI to simulate interactions can facilitate the development of competencies such as detailed anamnesis and diagnostic reasoning, which are critical components in TMD/OFP education, but which must always be integrated into clinical practice to be meaningful. Furthermore, simulation allows exposure to a variety of clinical cases that may be difficult to encounter in conventional clinical settings, enriching the educational experience and preparing future professionals to face diverse and complex challenges [29].

The use of GAI in educational environments has gained prominence due to its potential to transform the way learning occurs, especially in areas involving the acquisition of complex practical skills. However, students’ perception of the artificial nature of the interaction can significantly influence their engagement and the value they attribute to the activity.

The field of TMD and OFP is complex and challenging, requiring professionals to develop specific diagnostic and treatment skills and competencies. However, traditional training and clinical skill assessment resources are often limited, particularly when addressing subtle nuances and varied scenarios encountered in daily practice. These simulations, although limited to text‐based communication, focus on improving clarity, precision, and organization of diagnostic reasoning, preparing students for practical application in more complex contexts. Additionally, this project seeks to compare students’ questioning strategies and clinical reasoning with a gold standard established by TMD/OFP specialists, which is essential to ensure that diagnoses are achieved through appropriate means. This alignment ensures that the competencies developed by students correspond to recommended clinical practices and that the cognitive process is valued as much as the outcome. In this context, the application of GAI simulation, such as ChatGPT‐3.5, offers a unique and innovative opportunity to bridge this gap.

By simulating realistic and adaptive clinical interactions, ChatGPT‐3.5 can provide a safe and controlled environment for dental students to develop and refine clinical reasoning skills specific to TMD. Moreover, ChatGPT‐3.5's ability to deliver immediate and personalized feedback can facilitate the objective assessment of student performance, identifying strengths and opportunities for improvement. The use of GAI is proposed as a pedagogical strategy to complement traditional teaching by providing students with additional opportunities to practice communication and clinical reasoning skills in a controlled environment. This approach does not replace but rather enhances the learning that occurs in real clinical interactions. The underlying premise is that by preparing students for clinical interactions with a strong foundation in history‐taking and diagnostic reasoning, they will be better equipped to apply these skills in clinical practice, where physical evaluation and pain management are taught and practiced.

Therefore, exploring the applicability of this innovative technology in the field of TMD could not only enrich professional training but also improve the quality of care provided to patients with these conditions. To date, there are no publications in the scientific literature that have investigated the applicability of GAI simulation (ChatGPT‐3.5) as a tool for teaching skills and competencies related to clinical reasoning in the field of TMD. This pilot study aims to address this gap by evaluating the use of GAI‐based simulations for training and assessing clinical competencies in TMD/OFP.

Studies indicate that when students are aware they are interacting with AI, they may exhibit reduced engagement and adopt more superficial problem‐solving strategies, compromising the educational effectiveness of the tool [30]. Similarly, students’ motivation and the quality of their interactions are affected by the type of support offered by AI, as demonstrated by Kim et al. (2020), who emphasized the importance of careful pedagogical design to ensure meaningful interactions with artificial agents. Therefore, it is necessary to investigate strategies to minimize these behavioral biases, ensuring that AI can be used as an efficient educational tool aligned with the teaching needs in healthcare.

Building upon these findings, and as detailed in Table 1 and Figures 1 and 2, the quantitative analyses demonstrated that ChatGPT‐3.5 generated a higher volume of responses with greater informational density, while real patients promoted richer emotional exchanges and more dynamic clinical variability. This finding corroborates previous evidence showing that AI systems can deliver consistent and structured information, often with superior standardization compared to human interactions [19, 20]. In clinical education, this characteristic can support early‐stage learners by scaffolding their diagnostic reasoning and reducing ambiguity [25]. However, real patients, as demonstrated in this study, offered unique opportunities for students to develop adaptive questioning strategies and to manage uncertainty, both of which are crucial for authentic clinical reasoning [28, 31].

As evidenced by the correlations presented in Table 2, diagnostic accuracy was significantly associated with the presence of relevant information (*r* = 0.484, *p* = 0.007), but not with the total number of words produced. This reinforces the pedagogical importance of quality over quantity in clinical interviews, an aspect emphasized by previous research on clinical reasoning in dentistry and medicine [27, 31]. Although ChatGPT‐3.5 delivered more informational units, these were often highly structured and concise, while real patients presented more variable information delivery that required students to actively extract, synthesize, and interpret key data. These differences can have direct implications for the development of clinical reasoning skills, as the ability to navigate ambiguity is critical for competent patient care, especially in chronic and multifactorial conditions such as TMD [1, 2, 3].

**TABLE 2:** Point‐biserial correlation between diagnostic accuracy and quantitative/qualitative variables from clinical simulations.

Analyzed variable	Correlation coefficient (*r*)	*p* value	Interpretation
Relevant information	0.484	0.007	Significant positive correlation
Number of words	−0.386	0.035	Significant negative correlation
Empathy	0.299	0.108	Positive trend, not statistically significant
Reformulations	−0.274	0.143	Negative trend, not statistically significant
Number of responses	−0.191	0.311	Weak, non‐significant correlation
Clarity in communication	−0.068	0.720	No relevant correlation

*Note*: The point‐biserial correlation coefficient was used to examine the association between diagnostic accuracy (binary variable: correct = 1, incorrect = 0) and the performance scores in the simulations. Results with *p* < 0.05 were considered statistically significant. Data reflect pooled results from both simulation types: ChatGPT‐3.5 and real patient.

The qualitative content analysis presented in Table 3 further illustrates differences in communication dynamics. ChatGPT‐3.5 consistently used standardized expressions of empathy, such as “I understand how difficult this must be,” which can create a perception of emotional support but lack genuine affective depth [30, 32]. Real patients, on the other hand, expressed authentic emotional reactions grounded in personal experience, as illustrated by statements such as “This pain really makes me feel sad.” This distinction is critical for the development of relational and interpersonal skills, which are central to patient‐centered care [26, 27]. Empathy and emotional engagement are also particularly relevant for TMD and OP, where psychosocial factors play a substantial role in the onset and maintenance of pain [1, 2, 3].

**TABLE 3:** Comparative qualitative analysis between simulations performed by ChatGPT‐3.5 versus real patient, with interpretation and categorization according to Bardin (2011).

Category	Examples—ChatGPT	ChatGPT frequency	Examples—real patient	Real patient frequency	Interpretation according to Bardin (2011)
Empathy	Examples: “It is very difficult to deal with this.”; “It's annoying to go through this situation.”; “I feel bad when this happens.”	67	Examples: “I feel bad when this happens.”; “It's really annoying.”; “I feel sad with this pain.”	32	ChatGPT demonstrates formal and standardized empathy, whereas the real patient expresses genuine and affective empathy, closer to lived experience.
Clarity in communication	Examples: “I will explain it simply…”; “This means that…”; “Basically it occurs because…”	30	Examples: “It is a pain that I can't explain well.”; “It's more on one side than the other.”	16	ChatGPT's responses are more structured and didactic, whereas real patients’ responses may include hesitations and lack of clarity.
Relevant information	Examples: “I feel pain when I wake up.”; “The pain worsens with stress.”; “There was no recent trauma.”	231	Examples: “It hurts when I chew.”; “I wake up with jaw pain.”; “It started when I entered college.”	167	ChatGPT presents greater informational density, with complete and organized responses; real patients provide more contextual and subjective data.

*Note*: The categories were defined according to Bardin's (2011) Content Analysis: Empathy: expressions of acceptance, solidarity, or emotional validation; Clarity: objective and understandable structuring of responses; Relevant information: clinical and contextual data useful for diagnostic reasoning. The data show a higher frequency of occurrences in ChatGPT across all categories, especially in relevant information, whereas real patients demonstrate greater spontaneity and emotional richness but lower informational density.

Table 4 highlights differences in questioning strategies and clinical reasoning elicited by each simulation model. Real patient simulations resulted in a higher frequency of open‐ended questions and iterative reformulations by students, suggesting a more dynamic and adaptive diagnostic approach. This adaptability is an essential skill in clinical practice and has been identified as a key outcome of exposure to authentic patient encounters [28, 30]. In contrast, the structured nature of ChatGPT‐3.5's responses, while efficient, may reduce opportunities for students to practice managing ambiguity or incomplete information. This aligns with observations from Fryer et al. [30] that AI‐based interactions can sometimes lead to more superficial problem‐solving strategies when learners perceive the interaction as “artificial.”

**TABLE 4:** Characteristics of questions formulated by students after the simulations (ChatGPT‐3.5 vs. real patient): Open/closed classification, evolution of clinical reasoning, and interpretation according to Bardin (2011).

Aspect analyzed	ChatGPT‐3.5	Real patient	Frequency (ChatGPT vs. real patient)	Interpretation according to Bardin (2011)
Open questions	Examples: 1. “Can you tell me more about when the pain started?” 2. “How would you describe this pain?” 3. “In what situations does the pain worsen?”	Examples: 1. “What do you think is causing this?” 2. “What is this pain you feel like?” 3. “When did this start?”	42 (58%) vs. 29 (48%)	ChatGPT showed a higher proportion of open questions, with a more organized structure and formal language. The real patient used open questions spontaneously, but with less narrative exploration.
Closed questions	Examples: 1. “Is the pain on the right side?” 2. “Does it worsen when chewing?” 3. “Do you feel it when you wake up?”	Examples: 1. “Is it on this side?” 2. “Does it hurt when you chew?” 3. “Is it all the time?”	30 (42%) vs. 31 (52%)	Real patients tended to ask more closed questions, frequently used to confirm hypotheses. ChatGPT balanced open and closed questions in a more systematic way.
Evolution of clinical reasoning	Clear evidence of logical progression: started with general data (onset, duration, characteristics) and moved on to specific diagnostic hypotheses (TMD vs. odontogenic pain). Example: “When did the pain start?” → “Is it associated with chewing?” → “Do you feel clicking in the TMJ?”	Less organized alternation between open and closed questions, without a clear sequence. Example: “Is it on this side?” → “What is the pain like?” → “Does it hurt when chewing?”	—	ChatGPT demonstrated greater systematization and refinement of clinical reasoning, while the real patient showed spontaneity but a less clear and less logical diagnostic progression.

*Note*: This analysis considers the questions formulated by students as a direct consequence of the simulations conducted with ChatGPT‐3.5 or the real patient, allowing us to observe how the simulation model impacted the quality and structure of clinical reasoning. The classification of questions was based on Bardin's (2011) Content Analysis. It was evident that ChatGPT‐3.5 favored greater organization in the formulation of questions and in clinical reasoning, while real patients, although more spontaneous, alternated open and closed questions in a less structured manner, which may hinder students' diagnostic integration.

Table 5 synthesizes the unique advantages and limitations of each simulation model. ChatGPT‐3.5 offers scalability, standardization, and accessibility, making it particularly useful in educational settings with limited patient availability or when ensuring equitable learning opportunities across students is a challenge [19, 33]. Moreover, its capacity to deliver structured feedback can enhance formative assessments and align with competency‐based educational frameworks [27]. Real patients, on the other hand, bring authenticity, emotional variability, and non‐verbal cues that cannot yet be replicated by AI [17, 18]. These characteristics are essential for the development of higher‐order competencies, such as empathic communication and the ability to navigate clinical uncertainty [26, 31].

**TABLE 5:** Detailed comparison between clinical simulations using generative artificial intelligence (ChatGPT‐3.5) and real patients in teaching clinical competencies in TMD/OP.

Dimension	GAI (ChatGPT‐3.5)	Real Patients
Information density	**Advantage**: Complete and organized responses, providing a greater volume of relevant information (231 vs. 167). **Limitation**: Standardization may generate excessive data that is less consistent with real clinical variability.	**Advantage**: Contextualized responses, with information directly related to the patient's history. **Limitation**: Shorter and less organized responses, possibly omitting important data.
Clinical reasoning progression	**Advantage**: Clear logical structure, facilitating students’ clinical reasoning. **Limitation**: Linear responses, less challenging.	**Advantage**: Unpredictable sequence of responses stimulates critical thinking. **Limitation**: Progression can be fragmented, making case synthesis more difficult.
Clinical variability	**Advantage**: High standardization among simulations, useful for evaluation and structured teaching. **Limitation**: Low clinical variability, limiting students’ exposure to different patient profiles.	**Advantage**: Natural clinical variability, closely reproducing real cases. **Limitation**: Difficult to standardize cases, making comparisons between students more challenging.
Empathy and emotional expressiveness	**Advantage**: Responses include formal expressions of empathy. **Limitation**: Empathy perceived as procedural, less genuine, and lacking emotional depth.	**Advantage**: Genuine expression of empathy, generating greater connection with students. **Limitation**: Not all patients can clearly convey emotional support.
Non‐verbal communication	**Limitation**: Does not reproduce non‐verbal signals (facial expressions, pauses, tone).	**Advantage**: Presence of non‐verbal cues, enriching students’ communicational assessment.
Spontaneity / unpredictability	**Limitation**: Predictable and standardized responses, with little spontaneity.	**Advantage**: Unexpected situations enrich the learning experience. **Limitation**: May create additional difficulties for less experienced students.
Availability / accessibility	**Advantage**: Available 24/7, allows for numerous simulations in any educational context. **Limitation**: Dependent on students’ ability to ask appropriate questions to elicit relevant information.	**Limitation**: Dependent on patient availability and supervisor time to conduct the simulations.

*Note*: The table synthesizes the main advantages and limitations observed in each simulation model. Quantitative results (e.g., 231 vs. 167 relevant information units) were obtained from the analyses presented in Tables 1, 2, 3, 4. The evaluated dimensions reflect communicational, clinical, and logistical aspects directly related to teaching competencies in TMD/OP.

The broader context of these findings reinforces the need for hybrid educational models that leverage the complementary strengths of AI‐driven and real patient simulations. As reported in prior studies, hybrid approaches in simulation‐based education are associated with superior learning outcomes compared to single‐modality interventions, as they provide structured exposure to core clinical content while also cultivating adaptability through exposure to variability [28, 29]. The use of ChatGPT‐3.5 can also expand students’ exposure to a wider range of clinical cases, including conditions they may not encounter frequently in clinical rotations, thus enhancing the breadth of their training [2, 3].

In terms of applicability and perspectives, these findings support the integration of GAI‐based simulations into dental and medical curricula as a complementary tool to real patient encounters. The scalability and accessibility of ChatGPT‐3.5 allow for individualized, repeated practice in a controlled environment, which is essential for skill acquisition [19]. Future developments in multimodal AI platforms, incorporating voice, facial expressions, and non‐verbal behaviors, may further narrow the gap between AI‐driven simulations and authentic patient interactions [17, 18]. Longitudinal studies are needed to evaluate the impact of these hybrid approaches on skill retention, transfer to clinical practice, and patient outcomes. Additionally, exploring students’ perceptions of the authenticity and educational value of AI‐driven simulations may inform the refinement of instructional design and ensure meaningful engagement [30].

Study limitations: Despite its contributions, this study has limitations that warrant consideration. First, the exploratory nature and limited sample size reduce the generalizability of the findings. Second, the simulations were text‐based, which prevented the evaluation of non‐verbal communication skills, a critical dimension of clinical competence. Third, although the clinical cases were developed using the Delphi method to ensure diagnostic validity [22], AI‐generated responses remain influenced by training data and may not fully capture the complexity of real‐world clinical scenarios [16, 18]. Finally, the study focused primarily on immediate outcomes, such as diagnostic accuracy and communication patterns; future research should evaluate long‐term outcomes, including skill retention and transferability to real clinical practice. Mixed‐methods studies combining objective performance measures and qualitative data from learners and educators may provide a more comprehensive understanding of the pedagogical value of GAI‐based simulations.

## 7 Conclusion

The findings of this study show that GAI, through ChatGPT‐3.5, performs comparably to real patient simulations in teaching clinical competencies in TMD/OP, offering advantages such as greater information density, standardization, and accessibility. However, limitations related to emotional expressiveness, clinical variability, and the absence of non‐verbal cues indicate that GAI cannot yet fully replace real patient experiences, which remain essential for their authenticity, spontaneity, and emotional richness. These results reinforce that the integrated use of both models can enhance student training by combining the consistency and scalability of GAI with the communicational and clinical experience provided by real patients.

## Funding

This study was supported by Coordination for the Improvement of Higher Education Personnel (CAPES).
